# Systems biology analysis of drivers underlying hallmarks of cancer cell metabolism

**DOI:** 10.1038/srep41241

**Published:** 2017-01-25

**Authors:** Daniel C. Zielinski, Neema Jamshidi, Austin J. Corbett, Aarash Bordbar, Alex Thomas, Bernhard O. Palsson

**Affiliations:** 1 Department of Bioengineering, University of California, San Diego, La Jolla CA 92093-0412, USA; 2 Institute of Engineering in Medicine, University of California, San Diego, La Jolla CA 92093-0412, USA; 3 Department of Bioinformatics and Systems Biology, University of California, San Diego, La Jolla CA 92093-0412, USA; 4 The Novo Nordisk Center for Biosustainability at the University of California San Diego School of Medicine, University of California, San Diego, La Jolla CA 92093-0412, USA; 5 Department of Pediatrics, University of California, San Diego, La Jolla CA 92093-0412, USA

## Abstract

Malignant transformation is often accompanied by significant metabolic changes. To identify drivers underlying these changes, we calculated metabolic flux states for the NCI60 cell line collection and correlated the variance between metabolic states of these lines with their other properties. The analysis revealed a remarkably consistent structure underlying high flux metabolism. The three primary uptake pathways, glucose, glutamine and serine, are each characterized by three features: (1) metabolite uptake sufficient for the stoichiometric requirement to sustain observed growth, (2) overflow metabolism, which scales with excess nutrient uptake over the basal growth requirement, and (3) redox production, which also scales with nutrient uptake but greatly exceeds the requirement for growth. We discovered that resistance to chemotherapeutic drugs in these lines broadly correlates with the amount of glucose uptake. These results support an interpretation of the Warburg effect and glutamine addiction as features of a growth state that provides resistance to metabolic stress through excess redox and energy production. Furthermore, overflow metabolism observed may indicate that mitochondrial catabolic capacity is a key constraint setting an upper limit on the rate of cofactor production possible. These results provide a greater context within which the metabolic alterations in cancer can be understood.

Over the past decade there has been a revival of metabolic research in oncology^1^. In particular, two defining characteristics of cancer metabolism have received much attention: (1) an increased glucose uptake rate accompanied by secretion of lactate even in the presence of oxygen, known as the Warburg effect^2^, and (2) a high glutamine uptake rate essential for growth, known as glutamine addiction^3^,^4^,^5^,^6^,^7^. Despite the central role these traits play in the discussion of cancer metabolism, the drivers underlying these traits are still debated^8^. It is important to understand these drivers as cancer metabolism is likely to become a focus of chemotherapeutics development^1^,^3^,^9^.

The NCI60 cell line collection consists of 60 cancer cell lines that have been extensively used as a model to study characteristics of cancer cells over the past quarter of a century^10^,^11^,^12^,^13^,^14^. Notably, the metabolite uptake and secretion profiles for these lines were recently published^11^. When coupled to growth^15^ and cell size data^14^, these data provide the opportunity to study cancer metabolic functional states at an unprecedented scale by utilizing flux balance analysis (FBA)^16^. Fundamentally structured in the context of metabolic mass, energy and redox balance, FBA has been utilized successfully over the past decade as a method of data integration^17^ as well as a number of other applications^18^, including cancer metabolism^19^,^20^,^21^. Using FBA, we integrated available metabolic data to calculate metabolic flux states for the NCI60 panel. We then leveraged the differences in metabolic flux states across the NCI60 panel to identify drivers underlying two dominant features of cancer metabolism: the Warburg effect and glutamine addiction.

## Results

### Data-driven calculation of metabolic fluxes for the NCI60 cell line panel

**Figure 1:**
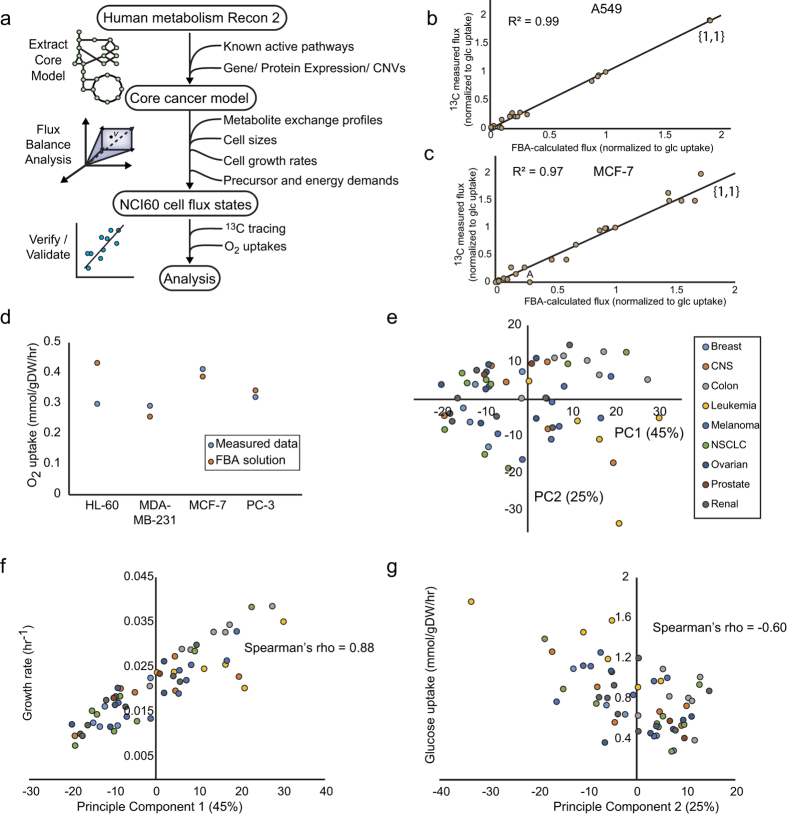
Data-driven characterization of the high flux backbone of the cancer metabolic network. (**a**) The workflow utilized in this study for the constraint-based calculation of metabolic flux states for the NCI60 panel using available data and a core metabolic model extracted from the global human metabolic network reconstruction Recon 2^22^. (**b**) Comparison of flux balance analysis results to a previously published ^13^C-labeled glucose tracing experiment on the A549 line. The computed flux solutions were corrected for a substantial difference in measured lactate secretion prior to comparison (Supplementary Data, Supplementary Methods). (**c**) Comparison of flux balance analysis results to a previously published ^13^C-labeled glucose tracing experiment on the MCF-7 line. The computed flux distributions were corrected for a difference in the active form of malic enzyme, which is forced by glutamine uptake to be the mitochondrial isozyme, consistent with other studies^36^ (Supplementary Data, Supplementary Methods). Point labeled ‘A’: Citrate shuttling to lipid synthesis is not observed in the data but is forced to be active in the model by lipid synthesis requirements. (**d**) Comparison of FBA-calculated and measured oxygen uptake rate data for a subset of the NCI60 lines. (**e**) Principle component analysis (PCA) of the flux balance analysis (FBA) calculated flux distribution of the NCI60 cell lines. (**f**) Correlation between the first principle component and the growth rates. (**g**) Correlation between the second principle component and glucose uptakes. Abbreviations: PPP- pentose phosphate pathway, TCA – tricarboxylic acid, ETC – electron transport chain, AA – amino acid, NEAA – non-essential amino acid, (**d**) NTP – (deoxy) nucleotide triphosphate.

First, we calculated metabolic reaction fluxes for each cell line in the NCI60 collection using FBA on a core cancer metabolic model constrained by measured cell line-specific uptake and secretion rates for 23 metabolites^11^, representing >99% of carbon exchange, as well as growth rates and cell sizes (Methods). This core model was derived from the human metabolic network reconstruction Recon 2^22^ and consisted of high confidence (i.e. highly expressed and/or essential) growth and energy pathways (Fig. 1a, see Methods). Genome-scale cell line-specific models were also constructed and evaluated (Supplementary Fig. 2), but inconsistencies between expression calls and known pathway function discouraged us from proceeding with their use (Supplementary Fig. 2d). Using reported karyotypes^23^, cell sizes^14^, and typical mammalian cell compositions^24^,^25^, we estimated cell-specific biomass compositions for each cell line^26^ (see Methods, Supplementary Data). These biomass compositions differ primarily in their fractional DNA content, as the karyotypes were the only reliable information on cell-specific biomass composition, while the protein fraction was assumed constant across cell lines. Cell line-specific protein fractions would likely increase the resolution of predicted cell line-specific flux states.

Flux balance analysis was run on each individually-constrained cell line with an NADPH maximization objective, as the other typical candidates for optimization objectives, which are ATP production and growth rate, were set based on measured data^15^,^25^. This objective also gave the best agreement with ^13^C tracing data (Supplementary Text and Data), and thus appeared to give the best estimate of fluxes for further analysis. ATP maintenance requirements were set based on data for a mouse *LS* line^25^, as a cancer cell line-specific value was not available. Furthermore, the glycolysis/pentose phosphate pathway ratio and the pyruvate dehydrogenase/pyruvate carboxylase ratios were constrained to 10% of the major pathway (glycolysis and PDH, respectively) based on previous ^13^C studies^27^,^28^ due to uncertainty and large model variation around these flux splits, and the glutamate and isocitrate dehydrogenases were assumed equally NAD versus NADP dependent. The uncertainty in flux splits is in large part due to the unknown non-biosynthetic NADPH demand due to oxidative stress, as well as unknown CO_2_ production, which together would provide additional knowledge on the activity of these pathways. As a sensitivity analysis, we varied these ratios between 1% and 20% of the major pathway and found that these ratios do not significantly impact the overall flux solution, with Pearson correlations for the same cell line greater than 0.99 being observed (Supplementary Data). This small impact appears to be due to the fact that the minor pathways control relatively small fractions of the cellular flux, and there may be alternate pathways that compensate for altered constraints on these ratios.

The flux states computed with the core model showed an excellent correlation with ^13^C data available for the A549 and MCF-7 lines^27^,^28^ (Pearson R^2^ > 0.95) (Fig. 1b,c) as well as measured oxygen uptake rates for a subset of lines with available data (average error of 17%)^29^ (Fig. 1d). As the calculation was partially fit to these flux states due to constraining certain flux splits (Methods), this agreement can be interpreted as verification rather than completely independent validation. However, this agreement is still important to obtain flux states that are more likely to be accurate for the purpose of downstream analysis. Notably, the oxygen data was not used as a constraint, and so agreement between predictions and data is especially encouraging. Furthermore, no cell line reached the upper limit we set on oxygen uptake of 2 mmol/gDW/hr, based on the observed range in mammalian cell lines^29^, indicating that oxygen uptake is not a governing constraint on these flux states given the objective function we used.

Principle component analysis (PCA) revealed that the metabolic states of the NCI60 lines are structurally simple (Fig. 1e, Supplementary Fig. 4), with large part of the variance explained by growth rate (Fig. 1f) and glucose uptake differences (Fig. 1g) (Supplementary Data). Among measured fluxes, glutamine uptake also makes a major contribution to the second principal component, as well as the dependent unmeasured exchange reactions for oxygen and CO_2_, suggesting that the second component can be interpreted as a total substrate intake component. Although there is no clear separation between distinct metabolic states apparent, examination of the states by tissue type shows some trends. For example, the leukemia-derived lines in the NCI60 tend to be characteristically fast growing with high substrate uptakes, while the colon-derived lines tend to be characteristically fast growing with low substrate uptakes.

### Comparison of amino acid uptakes to biomass precursor demands

**Figure 2:**
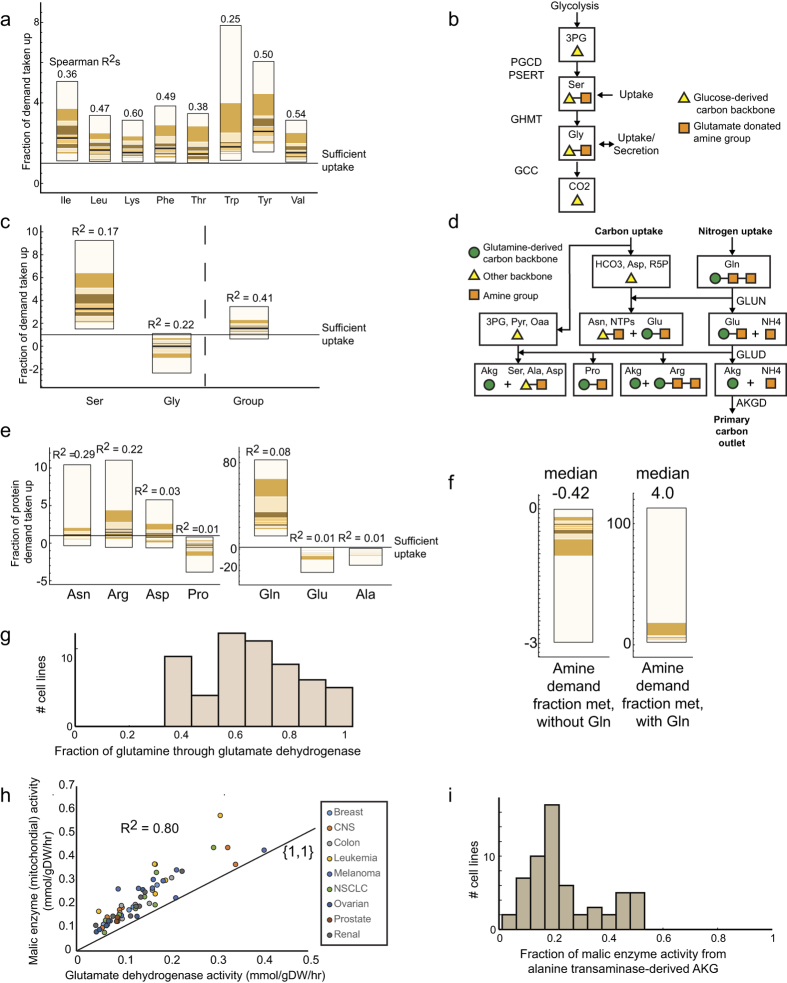
Amino acid metabolism is determined by protein synthesis demands and coupled to overflow metabolism. (**a**) Quantile plot of essential amino acid uptake compared with protein synthesis demand. Each band in the quantile plot represents the bounds of a particular quantile. Ten quantiles are shown. Listed above each bar is the Spearman correlation R^2^ of the amino acid uptake with biosynthetic requirement across lines. (**b**) Overview of serine and glycine synthesis pathway. (**c**) Quantile plots of the uptakes of serine and glycine relative to their demand from protein synthesis. (**d**) Overview of the metabolism of glutamine and related biosynthetic precursors, highlighting the role of glutamine in amine donation as well as its primary catabolic route through glutaminase and glutamate dehydrogenase. (**e**) Quantile plots of uptakes of glutamine-related amino acids compared to their protein synthesis requirement. (**f**) Overall amine balance due to protein synthesis requirement. (**g**) Fraction of glutamine uptake metabolized through glutamate dehydrogenase, with an average of 70%. (**h**) Correlation between glutamate dehydrogenase and the mitochondrial malic enzyme demonstrates that glutamine is primarily converted to pyruvate through malic enzyme, producing mitochondrial NADPH. (**i**) Fraction of malic enzyme flux due to alpha-ketoglutarate produced from alanine transaminase tied to alanine overflow, highlighting an alternate glutamine catabolic route.

Consistent with the dominance of the metabolic state by growth rate, we find that uptake of many amino acids are determined largely by growth requirements (Fig. 2a). Growth requirements, or demands on biomass precursors, are calculated by multiplying the coefficient for the metabolite in the biomass reaction by the growth rate. In particular, we found that essential amino acids, as well as tyrosine that can be derived from the essential amino acid phenylalanine, are taken up at levels only slightly greater than is required to meet protein synthesis demands. Additionally, uptake of each essential amino acid was found to be correlated with their biosynthetic demand across cell lines, consistent with previous findings^14^.

We identified a similar dominance by growth demands among the related amino acids serine and glycine (Fig. 2b). We found that serine uptake is consistently greater than its biosynthetic requirements while glycine secretion rate is highly variable (Fig. 2c). However, the combined uptake and secretion of the two amino acids matches their combined biomass synthetic requirements (Fig. 2c), as has been observed^14^. Additionally, the correlation of the uptake of the amino acid group as a whole with biosynthetic demand is higher than the correlation considering each amino acid separately. These results suggest that serine uptake acts as a reservoir to meet protein synthesis requirements for the two amino acids.

Looking then at the other major group of non-essential amino acids, which are linked through glutamate (Fig. 2d), we found the dominance of growth requirements to be less consistent. We found that, on average, asparagine, arginine, and aspartate are taken up about the levels of their biosynthetic demand, but with a lower correlation than in the case of the essential amino acids (Fig. 2e). However, glutamate, alanine, and proline were all consistently secreted, suggesting no direct connection between their uptake and growth requirement. Meanwhile, glutamine was taken up on average 32 times more than its biosynthetic requirement, an extremely high uptake rate demonstrative of the glutamine addiction phenotype (Fig. 2e).

### Determining the driver underlying glutamine uptake in the NCI60 cell line panel

To determine whether the high glutamine uptake is tied to the biosynthetic requirement for nitrogen groups as a whole, we looked at the amine balance with and without glutamine uptake considered (Fig. 2f). It was found that the cell lines are in a nitrogen deficient state without considering glutamine, with a median deficient of 42% of the demand. However, after considering glutamine uptake, nitrogen is in excess of biosynthetic demands by a median of 400% of the biosynthetic requirement for amino acids. This result suggests that glutamine uptake is necessary to fulfill the amine requirement for growth, but this requirement is not sufficient alone to explain the very high level of glutamine uptake in these cell lines. Other sinks for nitrogen such as nucleotide triphosphate synthesis were negligible. Thus, glutamine addiction is not sufficiently explained by the necessity to produce amino acid precursors for protein synthesis.

We then examined the metabolic fate of the excessive glutamine taken up by NCI60 lines. We found that glutamate dehydrogenase (GLUD) metabolizes an average 70% of glutamine taken up (Fig. 2g). However, α-ketoglutarate (αKG) produced from this reaction greatly exceeds the requirement for TCA metabolites and cannot be fully oxidized by the TCA cycle due to the structure of the pathway. To be fully catabolized, αKG produced from GLUD must exit the TCA cycle via malic enzyme (ME) to be converted into pyruvate for later oxidation. This pathway produces mitochondrial NADPH through GLUD and ME in a manner that scales with glutamine uptake. Simultaneously, an alternate pathway to glutamine catabolism through alanine transaminase exists that results in secretion of alanine, as is observed in these lines. These pathways can alternately be powered by glucose by producing oxaloacetate via pyruvate carboxylation, but this pathway is consistently measured as carrying relatively low flux^12^,^13^. We found that the primary route for glutamine catabolism is through this mitochondrial NADPH-producing malic enzyme pathway, as evidenced by correlations between glutamate dehydrogenase and alanine transaminase with malic enzyme activity (Fig. 2g,i). This pathway is primarily glutamine-driven rather than glucose-driven, and thus supports the idea that primary role of the high glutamine uptake is to produce mitochondrial NADPH, rather than to produce amine equivalents for protein synthesis.

### Calculating sources and sink of ATP production and drivers of the Warburg phenotype

**Figure 3:**
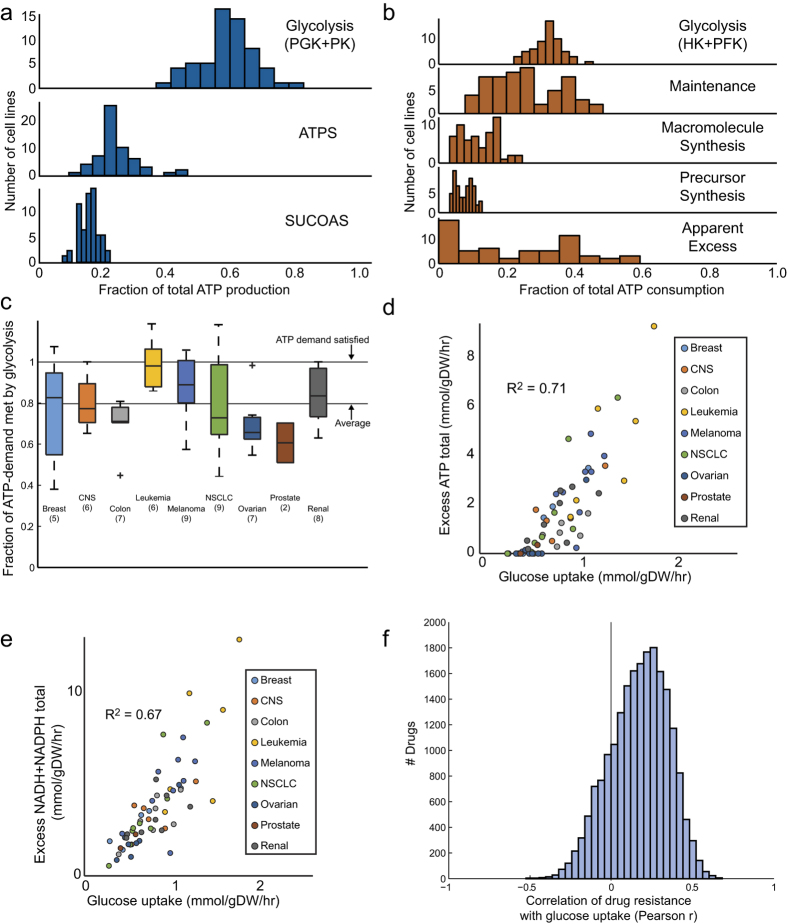
Glucose uptake drives ATP production in excess of known ATP utilization pathways. (**a**) Fraction of ATP production provided by glycolysis, the mitochondrial ATP synthase, and succinyl-CoA synthetase. It is observed that glycolysis provides on average ~60% of total ATP production, in line with previous measurements. (**b**) Fraction of ATP consumption by various cellular processes. (**c**) Fraction of cellular ATP demand satisfied by glycolysis. On average, 80% of total ATP demand is provided by glycolysis. (**d**) Comparison of glucose uptake with excess total ATP production. Although glycolysis satisfies 80% of cellular ATP demand, it produces only 60% of total ATP production, resulting in an excess of cellular ATP above growth and maintenance costs. (**e**) Comparison of glucose uptake with excess total NADH + NADPH production. As with ATP, the high rate of glycolysis results in excess NADH + NADPH production above what is required for biosynthesis. (**f**) Correlation between resistance to chemotherapeutics and glucose uptake. Glucose uptake correlates highly with excess energy and redox production, and simultaneously correlates broadly with resistance to chemotherapeutic drugs in a manner independent of cellular growth rate.

Next, we sought to apply a similar analysis to glucose metabolism, which is typically considered to be the primary source of energy in the cancer metabolic phenotype. Calculating the balance of NTP production and consumption (Fig. 3a,b), we determined that NTP production in the NCI60 lines has three main contributors: net glycolysis (mean 44% of production), ATP synthase (mean 35%), and succinyl-CoA synthetase (mean 21%) (Fig. 3a). Net glycolysis is defined as glycolytic production (phosphoglycerate kinase and pyruvate kinase) minus glycolytic consumption (hexokinase and phosphofructokinase). This split of NTP production is consistent with previous estimates^30^, which place glycolysis at a slightly lower fraction of NTP production but agree that oxidative metabolism provides the majority of NTP. Looking at NTP demands, it is apparent that macromolecule and precursor synthesis only make up a minor part of the cellular NTP demand. A larger fraction is consumed by maintenance functions, such as the Na-K pump, or by undetermined functions that may consist of variable additional maintenance costs or energy drains such as metabolic futile cycles.

We find that net glycolytic ATP production is set at a level that mostly (80%) satisfies the known growth and maintenance ATP requirements of the cell, with little apparent bias related to the cell line tissue of origin (Fig. 3c). However, due to the additional significant contribution of oxidative metabolism to ATP production, cell lines produce an excess of ATP, compared with known ATP demands^25^, that scales with glucose uptake (Fig. 2d). Additionally, there is an excess of redox production, in the form of both NADH and NADPH, over growth and energy demands that scales with glucose uptake as well (Fig. 3e). Thus, although glycolysis alone meets the growth requirements for ATP, the overall ATP and NADH energy requirement is exceeded due to the contributions from oxidative phosphorylation. To further quantify this excess, we calculated the minimum amount of glucose that could be taken up and still satisfy measured growth rate and known ATP demands (Supplementary Data). We calculated that the NCI60 cell lines on average could take up only 59% (standard deviation of 16%) of the actual measured glucose uptake and still satisfy known growth and energy requirements. Thus, it appears that there is additional metabolic flexibility due to high glucose uptake that appears to be driving cofactor production in excess of known demands.

Seeking to identify some relationship through which to understand this excess redox production, we correlated the glucose uptake with a large set of available data on the NCI60 lines, including gene expression, drug response, metabolomics, and proteomics. Remarkably, we found that higher glucose uptake broadly correlated with resistance to chemotherapeutic compounds within the NCI Developmental Therapeutics Program database, which contains sensitivity data for 20500 compounds (Fig. 3f)^10^. This correlation was independent of the association between drug sensitivity and cell growth rate. Notably, imatinib resistance is among the highest correlated to glucose uptake, and imatinib resistance has previously been shown to be associated with higher glucose uptake^31^.

### Topological consistencies link the dominant uptake metabolites in cancer metabolism

**Figure 4:**
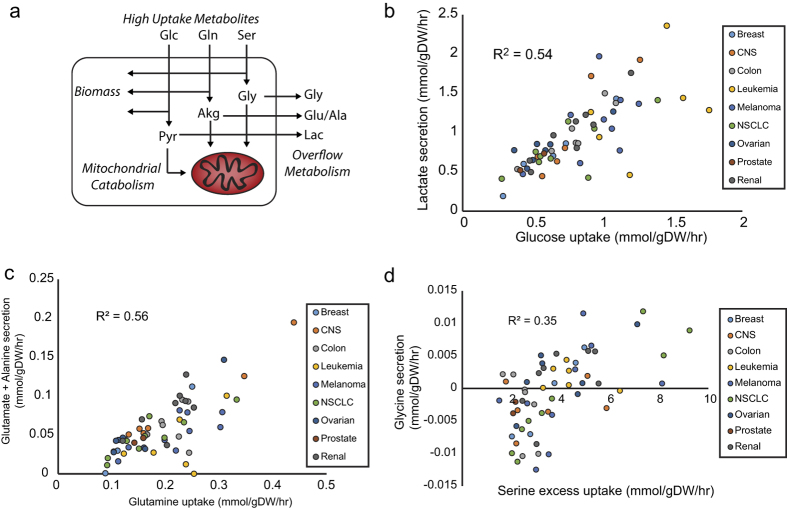
Overflow metabolism in high flux pathways in the NCI60 cell lines. (**a**) High uptake metabolites are coupled to overflow metabolism at branch points occurring above mitochondrial catabolic pathways. (**b**) Comparison of lactate secretion with the glucose uptake rate. The strong correlation suggests a direct relationship between the metabolite exchanges. (**c**) Secretion of glutamate and alanine vs the glutamine uptake supporting a role of the former two metabolites in metabolic overflow. Although alanine secretion individually is not significantly correlated with glutamine uptake, considering glutamate and alanine simultaneously as a secreted pool improves the correlation with glutamine uptake over glutamate alone, suggesting glutamate and alanine may behave somewhat as a secreted pool. (**d**) Glycine secretion vs excess serine uptake, calculated as serine uptake minus serine and glycine protein synthesis demand. A significant correlation is observed, suggesting a role of glycine as an overflow metabolite when serine uptake exceeds the requirement for protein synthesis and ability to metabolize glycine through the glycine cleavage chain.

Finally, looking at the question of what limits are placed upon the cancer metabolic phenotype, we make special note of a trend that was discovered among the three highest uptake metabolites: glucose, glutamine, and serine (Fig. 4a). We found that the uptake of each metabolite is coupled to secreted metabolites, which are lactate, glutamate, and glycine, respectively. First, glucose uptake is highly correlated with lactate secretion (Fig. 4b), a well-known feature of the Warburg effect. Second, glutamate secretion is significantly correlated with glutamine uptake, and consideration of alanine secretion as well improves this correlation (Fig. 4c). Finally, we found that glycine secretion increases as serine uptake increases above the cellular protein precursor requirement (Fig. 2d). Each of these secreted metabolites occur at branch points directly prior to a mitochondrial catabolic pathway. In the case of lactate (pyruvate) and glutamate, the pathway is the TCA cycle. In the case of glycine, the pathway is the glycine cleavage chain. The correlation of the excessive uptake of the high uptake metabolites with the secretion of coupled overflow metabolites may suggest that mitochondria are insufficiently able to catabolize the taken up metabolite. This could suggest that mitochondrial catabolic capacity is a governing constraint limiting the fast growing metabolic phenotype.

## Discussion

Taken together, the results of this structured analysis of disparate data types suggest a fundamental structure to cancer cell metabolism across multiple tissue types where biosynthetic demands are primary determinants of much of the metabolic phenotype. However, glucose and glutamine uptake rates scale above the biomass requirement resulting in an excess of redox and energy cofactor production beyond biosynthetic and energy requirements. These results suggest an interpretation of the Warburg effect and glutamine addiction as drivers of metabolic stress resistance. The role of these high uptake pathways on sustaining stress resistance has been demonstrated before in individual cases^32^. The idea that the metabolic shift from high yield to low yield metabolism, such as in the Warburg effect, may be due to competition for redox cofactors has been suggested elsewhere in the literature^33^.

A number of other ideas have been put forth on the drivers underlying the Warburg effect and glutamine addiction. We used the abundance of available data on the NCI60 cell line panel with the rigorous metabolic network analysis framework to evaluate whether these alternate hypotheses may also be promising in explaining the origin of the Warburg effect and glutamine addiction. First, we looked at explanations put forth for origin of the Warburg effect. We examined the hypothesis that the shift to aerobic glycolysis occurs to supply the cell with biosynthetic precursors^7^, such as serine and UDP-glucose, required for synthesis of macromolecules. Metabolic models can simultaneously reconcile biosynthetic precursor and energy needs for growth^34^. We found that in no cell line was production of a glucose-derived biosynthetic precursor the limiting factor for growth (Supplementary Fig. 3d). In fact, synthesis of glucose-derived biosynthetic precursors quantitatively accounts for only a small fraction (<10%) of glucose uptake in all cell lines (Supplementary Fig. 1a). One of several essential amino acids is the growth-limiting factor in all but two lines, in which ATP is limiting (Supplementary Fig. 3d). Furthermore, as we detailed above, serine is taken up sufficiently for both serine and glycine biosynthetic requirements. Taken together, the quantitative assessment of the need for metabolic resources afforded by the network models argues against the notion that biosynthetic precursor production is a driving factor underlying the Warburg effect. While variance in biomass composition, which we cannot easily account for, will affect these values, the calculated fraction of glucose going to biomass precursors seems too small for such variance to affect these conclusions.

Then, we examined a recently proposed hypothesis that the Warburg effect serves to reduce spatial constraints due to macromolecule crowding by shifting from high protein-requiring oxidative phosphorylation to primarily glycolysis^4^,^35^. Much like the balance between redox production, growth, and mitochondrial catabolism proposed in this work to explain the Warburg effect, this alternate hypothesis can be seen as driven by a ‘trade-off’ relationship, where an apparently sub-optimal metabolic phenotype is adopted due to certain cellular limitations. To examine this hypothesis, we analyzed published quantitative proteomics data for the NCI60 collection^13^. We found that metabolic proteins account for approximately 20% of the proteome, while 10% of proteins within metabolism are associated with the TCA cycle and oxidative phosphorylation (Supplementary Fig. 1b). Thus, within the NCI60 cell line collection, only approximately 2% of the proteome is associated with oxidative phosphorylation, a small fraction that does not support the notion that the reduction of oxidative phosphorylation can significantly reduce macromolecule crowding. In a subsequent analysis, we hypothesized that if the Warburg effect is driven by a macromolecule crowding constraint, then a metric of macromolecular crowding should be inversely correlated with the degree of aerobic glycolysis within the line. We correlated the protein density within the NCI60 lines with two metrics of the Warburg effect based on calculated metabolic flux distributions. We did not find any positive association between the metrics and protein density however, further arguing against any functional connection between the degree of macromolecule crowding and the origin of the Warburg effect, at least at the whole cell level (Supplementary Fig. 1c).

We then examined existing hypothesis for drivers of glutamine addiction. First, we examined the idea that the role of glutamine’s high uptake is to provide the pool of TCA metabolites^36^. Glutamine has been shown to be the primary source of the TCA metabolite pool^36^. However, from a flux balance perspective, it appears that this cannot quantitatively explain the high level of glutamine uptake. Assuming a steady state, the TCA metabolite pool will gradually drain due to growth dilution. The rate of dilution is the current concentration of the metabolite multiplied by the growth rate. Assuming a concentration on the high end of 1 mM, a growth rate of 0.02 hr^−1^, a cell mass of 0.3 ng, and a cell volume of 2 pL, which are approximate averages for the NCI60 lines, each TCA metabolite would need to be replenished at a rate of 0.13 uM/gDW/hr. However, glutamine uptake is an average of 0.19 mM/gDW/hr for the NCI60 cell lines, three orders of magnitude higher. Thus, replenishing the TCA metabolite pool only demands a small fraction of glutamine uptake. Although glutamine enters the TCA cycle and provides TCA metabolites, this does not sufficient support that this is the primary reason for the high glutamine uptake, since the majority of glutamine cannot stay within the TCA cycle.

Second, it has been proposed that the cleavage of ammonia from glutamine and glutamate by glutaminase and glutamate dehydrogenase can serve as a pH buffer to mitigate the acid production due to lactate and CO_2_ production^6^. We contend that the production of NH_4_ can do very little to serve as a pH buffer for two reasons. First, neither of the enzymes glutaminase and glutamate dehydrogenase consume a proton directly as a reactant. Second, these reactions produce NH_4_ with the proton already bound, preventing the molecule from scavenging further protons produced from other processes. In line with this, we observe no correlation between glutamine uptake and any acid production metrics, including secreted protein, secreted lactate, and secreted CO_2_. However, this argument is flux-based and says nothing about a potential regulatory role that ammonium production may have.

One surprising result of this study was the broad correlation of glucose uptake with drug resistance in the NCI60 cell line panel. We note that this correlation was found between glucose uptake in per mass units as opposed to per cell units, which are the original units reported^11^. There is an important difference in the interpretation of metabolic flux in these units. Because the cells are different masses, the fluxes per mass and per cell are not expected to correlate and any observed correlations with other variables such as drug resistance could have different interpretations. Two flux states in per cell units could potentially be the ‘same’ flux state in a qualitative sense, i.e. in terms of relative pathway usage, but simply scaled by the size of the cell; however, differences in flux in per mass units between cell types indicates a qualitative shift in pathway preferences within metabolism rather than a general scaling effect. Another study has shown a correlation of cell volume with cell mesenchymality, as measured by expression of the marker vimentin^14^. Also, cell volume is substantially correlated with glucose uptake in per cell units (Pearson R^2^ = 0.39). Thus, it may be conjectured that the mesenchymal nature of the cell could be the underlying origin of the observed drug resistance in high glucose uptake cell lines. However, while we did verify a correlation of vimentin with cell volume (Pearson R^2^ = 0.22), we found that mesenchymality as estimated by this marker does not correlate with drug resistance in these lines, with a median Pearson correlation coefficient r of −0.04, indicating a non-significant relationship opposite of the hypothesized direction of correlation.

Investigating into the relationship between glucose uptake and drug resistance, we hypothesize that the excess redox and energy cofactor production by high glucose uptake lines provides a measure of resistance to metabolic stress imposed by chemotherapeutics as a part of their mechanism of action. The role of metabolic stress in the mechanism of action of chemotherapeutics is becoming increasingly well established in the literature. We take a look at two such metabolic stresses: ATP depletion and oxidative stress. ATP depletion has long been targeted as a possible adjutant strategy for increasing the effectiveness of chemotherapy^37^,^38^. For example, one study showed that intracellular levels of ATP are critical for drug resistance in colon cancer cell lines^39^. Another study showed that cellular bioenergetic propensity was correlated with decreased sensitivity to sorafenib^40^. However, in the latter study, activation of oxidative phosphorylation, accompanied by an increase in ATP levels, sensitized the cells to sorafenib, potentially indicating that ATP is not the critical factor governing sorafenib sensitivity. NADPH is a key cofactor that is responsible for combatting damage by reactive oxygen species within the cell by maintaining the reduced state of cofactors pools such as glutathione that can neutralize reactive oxygen species. Oxidative stress has been shown to sensitive tumor cells to chemotherapeutics^41^. One study showed that inhibition of G6PDH, the committed step of the pentose phosphate pathway that is regulated by the NADPH/NADP ratio, sensitizes cisplatin-resistant cancer cells^42^. Previous work has shown that the PKM2 isozyme implicated in inducing the Warburg metabolic phenotype also responds to reactive oxygen species by increasing oxidative stress resistance through increasing pentose phosphate pathway flux^43^. By investigating the top correlated drugs, we also identified a number of drugs that had previously been shown to induce apoptosis via an oxidative stress-dependent mechanism (Supplementary Data). Given this evidence, it seems possible that ATP depletion and NADPH depletion can both be sensitizers of chemotherapeutics in different contexts; however, the molecular mechanisms underlying this relationship appear yet to be elucidated in the majority of cases.

We note that stress resistance has been associated with the appearance of aerobic glycolysis in other contexts than malignant transformation. Yeast has been shown to shift toward anaerobic glycolysis during normal growth in order to minimize energy demands and stresses associated with aerobic metabolism^44^. Immune T cells have been shown to exhibit aerobic glycolysis^45^, and T cell activation has been shown to be ROS-dependent^46^. Aerobic glycolysis also appears in stem cells, where maintaining a reductive state appears to be important for the potential to self-renew^47^,^48^. Virus infection has also been shown to induce the Warburg effect in infected cells^49^,^50^, and this shift has been shown to counteract virus-induced reactive oxygen species production^50^. Additionally, the brain has been shown to exhibit various levels of aerobic glycolysis, with possible implications in Alzheimer’s disease^51^, although ties to oxidative stress have not been demonstrated yet to our knowledge.

This work presents a cohesive framework underlying high flux metabolism in cancer cell lines. Several natural questions arise regarding the universality of the presented metabolic structure. First, whether these results apply to non-cancer rapidly growing cells is unknown; however, it has been shown that the Warburg effect appears in many physiological contexts, and thus it appears likely that some of the structure may be consistent between cancer and healthy growing cells. The overflow metabolism we identify within the serine and glutamine groups has been shown in stem cells^52^ and as a regulatory mechanism for pyrimidines in *E. coli*^53^. Having a sole high uptake amino acid may simplify the coordination required to control amino acid uptake at levels required by biosynthetic demand.

Second, the relevance of cancer metabolic hallmarks identified in cell culture to *in vivo* cancer metabolism has been called into question. For example, certain cancers do not exhibit the Warburg effect *in vivo*, and other cancer subtypes show an importance of *de novo* serine synthesis^54^,^55^ whereas serine uptake is dominant in the cancer cell line data. Thus it appears that there are likely multiple basic metabolic states that cancer can adopt, and understanding the differences in this structure is likely a fruitful avenue for further work. Furthermore, the results presented are for unperturbed cells growing in culture, and understanding how the basic structure changes under metabolic perturbation such as hypoxia will be important as well. Finally, similar analysis of primary tumors and tumor progression, and comparative analysis of subtypes (e.g. AML1 though 5) would lead to a finer grained view of cancer metabolism of clinical importance. Furthermore, within a tumor or tumor microenvironment it has been established that sub-populations of cells exist with different metabolic states^56^. The relevance of metabolic traits of metabolic traits of cancer cells in culture to the metabolic states of heterogeneous cancer cells in a tumor requires further investigation. However, we remarkably noticed no separation in metabolic state by cancer tissue of origin, suggesting that the metabolic framework we describe is a highly conserved feature of a rapidly growing state in culture. The results presented may serve as a baseline for understanding metabolic homeostasis and global regulation of metabolism.

Another area requiring further study is the role of lipid metabolism in the cancer metabolic phenotype as described in this work. There was very little data on lipid uptake in the exometabolomic study used to constrain metabolite uptakes^11^, thus, for example there is no way to know whether beta oxidation is significantly active in these lines. Using standard cellular lipid compositions (Supplementary Data), we estimated sinks of cytosolic NADPH and found that fatty acid synthesis and cholesterol synthesis are the dominant known requirements for NADPH (Supplementary Fig. 5a). However, there may also be additional unknown requirements for NADPH to combat oxidative stress. Elucidating the interplay of oxidative stress, lipid homeostasis, and NADPH would help to better understand the apparent excess of cofactor production occurring in these cell lines.

This study attempted to integrate various qualitative and quantitative data sources to identify general principles underlying the metabolic phenotype in cancer cell lines. A key issue in such a study is the quality of data used. In the course of the investigation, we identified several data sets that we found to be of insufficient quality to include for analysis. Other data, such as the exometabolomic data^11^ and cellular protein content^14^, required additional curation to use with confidence. Even still, it is important to examine any hypothesis from multiple angles and using multiple data sets to minimize the chance of data issues to cause false conclusions to be drawn. For example, in the case of examining the possible role of macromolecular crowding in the Warburg effect, we used two independent data sets on the cell protein content: one on high throughput proteomics^13^ to estimate the quantitative fraction of the proteome affected by the shift between the aerobic and Warburg phenotype, and one on total cell protein content^14^ to determine whether there was a correlation between the apparent intracellular crowding as estimated by cell protein density and the degree of Warburg effect manifested in the cell line. In this case, neither data set supported protein crowding at the whole cell level as a driving factor underlying the Warburg effect.

Another technical area of a data analysis study such as this is determining the desired complexity of the relationships investigated. In the case of this study, we largely looked at simple correlations between variables in an effort to maximize the interpretability of any findings. However, more complex grouping of variables and non-linear relationships could be found with more advanced statistical and machine learning methods. We attempted this approach initially, but found that showing the interpretability and statistical significance of complex signatures was a challenging task, especially faced with the difficulty of multiple hypothesis testing in cases where hundreds of thousands of data points from various data types are available, as are for the NCI60 cell lines. However, it is quite likely that more complex relationships are yet to be discovered in the data and this may prove a useful area of investigation.

This work involved the use of a simplified ‘core’ model of metabolism derived from human Recon 2. This model consisted of pathways involved in core energy metabolism and production of known biomass precursors. The use such a model is always at risk of ‘missing’ a key pathway that could affect model predictions. In the case of this study, our calculations were predominantly based on measured growth and metabolite uptake and secretion data. We attempted to include any pathways that had been measured to be active in prior ^13^C tracing studies of which we were aware. Still, there are a number of pathways, such as lipid catabolism as mentioned, for which we did not have any ‘functional’ (i.e. flux) data. Thus, the conclusions in this work are subject to be modified if pathways carrying a large amount of flux are later characterized, for example lipid metabolic, protein glycosylation, and epigenetic methylation and acetylation are relatively under-characterized from a quantitative flux perspective.

The results of this study suggest two avenues of inquiry. First, while the NCI60 cell line panel is historically important due to its high degree of characterization, it will be important to determine whether the metabolic structure described in this study is conserved in primary tumors. Second, as the overflow metabolites (lactate, glutamate/alanine, glycine) coupled to high uptake metabolites (glucose, glutamine, serine) occur at branch points upstream of catabolic pathways in the mitochondria, we hypothesize that mitochondrial catabolic capacity may be a limiting constraint preventing complete substrate catabolism in high uptake metabolism in cancer cells. Investigation of the role of mitochondrial catabolic capacity in regulating the cancer metabolic phenotype may thus be a fruitful approach. The broad range of important metabolic pathways that lie in the mitochondria is becoming increasingly appreciated^57^. Further elucidation of the fate of the excess redox and energy produced in cancer cell lines should consolidate our fundamental understanding of the hallmarks of cancer metabolism established here, and guide efforts at developing new chemotherapeutic strategies.

## Methods

### Construction of a core cancer model

To construct a minimal model, we included reactions necessary for biomass formation and primary metabolite catabolic pathways, while excluding anabolic pathways not associated with core biomass precursor production as well as secondary catabolic pathways. The primary distinction to be made was thus determining which reactions belong to primary and secondary catabolic pathways. This classification was performed algorithmically with manual justification based on (1) literature evidence^12^,^29^,^30^ and (2) feasibility based on modeling results.

The following pathways were included: (1) pathways required for synthesis of biomass precursors, (2) core energy metabolism (glycolysis, the pentose phosphate pathway, the TCA cycle, the electron transport chain, and the malate aspartate shuttle), (3) pathways previously shown to be active in cancer cell lines under normal conditions^12^,^29^, (4) cofactor transfer reactions for NTPs and redox groups, (5) pathways involved with essential and/or high uptake/secretion metabolites, (6) pathways involved in catabolism of essential and/or high uptake/secretion metabolites, (7) pathways involved in cofactor regeneration and small metabolite processing/transport for cofactors/small metabolites produced in pathways gathered from the previous steps. Pathways that were excluded as a result of these criteria tend to have the following properties: (1) pathways that have unknown activity but due to small flux can be thought of as noise in approximations (e.g. glycosylation, EAA anabolic pathways, beta-alanine), (2) pathways removed because not measured to be major contributor to catabolism of high uptake metabolites (GLUDC, SERHL and methylglyoxyl), (3) pathways that use high uptake metabolites only as cofactors (e.g. cysteine production from serine, where cysteine was not measured).

In general, we found that the addition of further pathways beyond those defined above would have to be accompanied by additional assumptions on uptake rates or cellular demands related to these pathways that would have made the modeling useless for practical differentiation between metabolic function across cell lines. As an example, we look at the possibility of including the methionine pathway. We found that modeling this pathway is difficult because the pathway is adjacent to cysteine metabolism, and the uptake of cysteine was not measured. Cysteine requires methionine to synthesize unless cysteine is directly taken up. However, not enough methionine is taken up to satisfy the biomass requirement for both amino acids. Thus, when we included this pathway along with biomass demands on methionine and cysteine, none of the cell lines were able to meet biosynthetic demands. The possibility of including this pathway was originally examined when we were defining the scope of the core model, and we discovered that it was not possible to model without making up an artificial cysteine uptake, which was problematic.

Other pathways are difficult to include because their composition in biomass is unknown. Specifically, the glycosylation pathway might be very interesting to study across the NCI60 cell lines; however, without glyco-profiling these NCI60 cell lines, we have no basis for any differential production across the cell lines, analogous to differential DNA production based on karyotype for example, and so any modeling we could do of these pathways is extremely limited. Thus, we only are able to include an approximation of the carbohydrate macromolecule content of the cell dry weight in the biomass without any meaningful specification of the particular macromolecule form of those carbohydrates.

Based on this approach, a core metabolic network of 382 reactions (Supplementary Data) was extract from the latest human metabolic network reconstruction^22^. Gene^58^ and protein expression analysis^13^ showed that this core set of reactions is highly conserved between cell lines, with the majority of reactions being highly expressed in all NCI60 lines (Supplementary Fig. 3b). The core metabolic network was used to account for observed physiological functions, i.e. growth and measured uptakes, and minimize the influence of pathways with an unknown functional status on the network’s flux state.

### Cell-specific biomass composition determination

Cell biomass is composed of protein, lipids, DNA, RNA and small molecules, in weight fractions determined by cell composition studies^26^. Average protein amino acid composition was taken from literature^24^,^59^,^60^. Approximate DNA deoxyribonucleotide composition was set based on genomic base frequency taking into account the karyotype of the NCI60 lines^23^. RNA ribonucleotide composition was determined based on measured mass fractions^24^,^59^,^60^. Lipid composition was set based on measured lipid composition for high concentration lipids. Small molecule weight fractions were determined for several high concentration non-essential metabolites using literature concentrations, using a typical cell dry weight of 0.2 ng/cell and cell volume of 2 pL/cell when unit conversions were necessary. Without any specific information on protein fraction of cell dry weight across the NCI60 cell lines, we assumed a constant value of 70% of dry weight. For the remainder of the non-protein cell dry weight, we used the measured karyotype of the cell lines to estimate the DNA content, e.g. tetraploid cell lines would have twice the mass fraction of DNA as diploid cell lines. The remainder of the cell biomass that was not accounted for by protein or DNA was distributed based on measured values among lipids and RNA.

We chose to set the macromolecule weight fractions to be constant between lines. Previous studies show minimal variance between macromolecule weight fractions for particular types of cells, such as hybridoma cells and Chinese hamster ovary cells^24^,^59^,^60^. Other cell types, such as liver cells, may have significantly different macromolecule weight fractions, but cell lines derived from such tissues are not present in the NCI60 panel. Also, although cell composition has also been reported to change across growth conditions^61^, the NCI60 panel was subject to uniform growth conditions in the studies generating the data used in this study. Furthermore, there is the question of whether cell composition changes with cell size. One study showed that doubling of cell size resulted in approximate doubling of respiration, suggesting the protein content scales proportionally to size^62^. Also, as volume changes, the cell surface area (SA) to volume (V) ratio changes, and thus it is possible that the lipid weight fraction of the cell changes as well. However, compartment size has been shown to be approximately linearly correlated with total volume^32^, and ER membrane alone is reported to be over 10 times the fraction of the total membrane as the cytoplasmic membrane^63^, suggesting SA/V differences mean little in terms of lipid requirements. Thus, we assumed that the macromolecule composition was invariant across cell lines, although cell sizes differ. Protein content and cell volume data for the NCI60 was recently published. However, this data was insufficient to set cell-specific biomass macromolecule weight fractions, as the cell dry weight was not measured. We examined the impact of varying the protein fraction from 0.7 to 0.6, and found that the correlation between calculated flux states was greater than 0.99 for the same cell line (Supplementary Data). However, altering the protein fraction further caused the flux balance analysis problem to become infeasible for a subset of the cell lines, as the altered protein dry weight content also changes the amino acid requirement for these cell lines, and since essential amino acids are limiting in a subset of the lines, this limitation can become constraining, resulting in the uptake and growth rate constraints becoming infeasible.

To determine the cell-specific dry weights, we integrated cell volume data with the uptake rates as follows. First, the amount of biomass sustainable by each cell was determined by maximizing the growth through each line using FBA while constrained by measured uptakes in per cell units. Then, this sustainable biomass was corrected using measured protein content data as follows. If the sustainable protein, taking protein as 0.70 of total cell dry mass, is less than the measured protein, a value of 95% of the sustainable protein measurement was used as the estimate of cellular protein. This was done because the measured protein could not be sustained by the measured uptake rates, which we assumed was due to error in the measured protein. Measured protein was assumed to be the greater source of error because the measured uptake rates are highly correlated and there was no general bias of sustainable protein being greater or less than measured protein. Also the measured protein showed a relatively low agreement with cell volumes (Pearson R^2^ = 0.23) and we observed certain spurious data points causing concern. For example, the SR line was reported to have a protein content of 0.021 ng/cell, which given the reported cell volume and average protein density would result in a dry weight fraction of protein of approximately 0.08, which is substantially lower than measured values around 0.7. Volume measurements were based in microscopy, and thus were seen as less error prone than protein content measurements which require cell count estimation, which can be a significant source of error. When sustainable protein was greater than measured protein, the measured value was used to correct the sustainable protein, using the formula m_estimate_ = m_measured_ + 0.25*(m_sustainable_ − m_measured_). This formula was chosen based on resulting agreement with cell volume data. The correlation of estimated protein content with cell volume (Pearson R^2^ = 0.60) was higher than either measured protein (Pearson R^2^ = 0.23) or sustainable protein (Pearson R^2^ = 0.52).

### Curation of exometabolic data

Published metabolite uptake and release (exometabolomic) data on the NCI60 lines was re-processed in a semi-automated process. The original dataset was processed by correcting for drift in the peak area standardization across runs by a linear L1 regression of blank media standards. However, upon detailed inspection of the drift for different metabolites, it was apparent that the drift was highly non-linear for some metabolites. The effect of applying a linear approximation in these non-linear cases was that metabolite uptakes were significantly mis-represented, and in some cases, metabolites were actually not exchanged substantially at all, once a non-linear drift correction was applied. We manually created non-linear approximations of drift for each metabolite in Mathematica based on the media standards for each metabolite. We applied these non-linear corrections to the raw data to recalculate the metabolite uptake and release profiles.

### Calculation of metabolic flux states

To calculate metabolic flux states, a stoichiometric model of central and growth metabolism was extracted from the latest global human metabolic network reconstruction, Recon 2^64^. Then, for each cell line, this model was constrained by cell line-specific data, including growth data^15^, metabolic uptake and secretion profiles^11^, and an estimate of cell mass based on sustainable biomass that was validated against measured cell sizes^14^. ATP costs due to cellular maintenance functions was set to be 1.07 mmol/gDW/hr based on measurements^25^. Twenty-three metabolites had uptakes constrained by measured data, consisting of glucose, lactate, and amino acids, which accounted for greater than 99% of observed metabolic exchange fluxes in the NCI60 cell line data set^65^ (Supplementary Fig. 3c). The amino acids histidine and cysteine were not analyzed due to lack of uptake measurement, while methionine was excluded due to its close relationship with cysteine. Because methionine can be used for cysteine biosynthesis, the lack of cysteine measurement caused us to be unsure of the amount of methionine used to protein synthesis directly versus cysteine biosynthesis. Furthermore, two flux splits, that between glycolysis and the pentose phosphate pathway (PPP), and between pyruvate dehydrogenase and pyruvate carboxylase, were constrained to be 90/10 each, based on an approximate average of previous ^13^C tracing studies performed on the NCI60 lines (Supplementary Data). The A549 cell line had a reported value of 16% of glucose uptake going to the PPP, while the MCF7 cell line had a reported value of 5% of glucose uptake going to the PPP. Furthermore, the isocitrate and glutamate dehydrogenases were assumed to equally utilize NAD versus NADP as substrates.

Metabolic flux states were then calculated by solving the flux balance analysis problem:


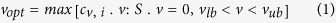


where ***S*** is the stoichiometric matrix, ***v*** is the reaction flux vector, ***α*** and ***β*** are vectors for the lower and upper bounds of the reactions, and **c**_***v**,**i***_ corresponds to the imposed reaction objective for each *i*^*th*^ sink reaction. A number of objectives were evaluated, including maximization and minimization of ATP production, maximization and minimization of cellular redox production, and minimization of overall cellular flux, a proxy for minimizing the proteomic cost of enzyme synthesis (Supplementary Data). We found that maximization of mitochondrial NAD(P)H gave the best agreement with ^13^C tracing data^12^,^13^, verifying the solutions on a subset of the NCI60 lines. This reaction is defined as NADPH − > NADP + H, for compartment-specific metabolites, and represents the irreversible loss of a redox ‘charge’ on the NADP moiety, forcing its replenishment by metabolic pathways to satisfy mass balance. Other redox production reactions that we evaluated, e.g. for NADH, were defined in an analogous manner with metabolites specific to the compartment.

To summarize the flux calculation procedure, we:

1. Started with a core metabolic model of a cancer cell, as defined elsewhere in the manuscript.
2. Constrained uptake fluxes based on cell line-specific measurements and certain non-cell line-specific approximations, specifically ATP maintenance, oxygen uptake, and certain flux splits.
3. Created a partially cell line-specific biomass function for each cell line based on the measured karyotype of each cell line.
4. Constrained growth rate based on cell line-specific measurements.
5. Solved a flux balance analysis optimization problem with an NADPH production objective.

The specific constraints used are provided in the “FBA constraints” table in the Supplementary Data.

## Additional Information

**How to cite this article**: Zielinski, D. C. *et al*. Systems biology analysis of drivers underlying hallmarks of cancer cell metabolism. *Sci. Rep.* **7**, 41241; doi: 10.1038/srep41241 (2017).

**Publisher's note:** Springer Nature remains neutral with regard to jurisdictional claims in published maps and institutional affiliations.

## Supplementary Material

Supplementary Text
