# Supplementary material for: Systems biology analysis of drivers underlying hallmarks of cancer cell metabolism
Source: Sci Rep. 2017 Jan 25;7:41241. doi: 10.1038/srep41241 (PMC5264163; doi:10.1038/srep41241)
Supplement: Supplementary Text [file srep41241-s1.pdf]

Supplementary Materials for

Systems biology analysis of drivers underlying hallmarks of cancer cell metabolism

Daniel C. Zielinski[1], Neema Jamshidi[1,2] Austin J. Corbett[1], Aarash Bordbar[1, 3], Alex Thomas[4,5,6], Bernhard O. Palsson[1, 6] *

[1] Department of Bioengineering, University of California, San Diego, La Jolla CA 92093-0412

[2] Institute of Engineering in Medicine, University of California, San Diego, La Jolla CA 92093-0412

[3] Current Address: Sinopia Biosciences, San Diego, CA

[4] Department of Bioinformatics and Systems Biology, University of California, San Diego, La Jolla CA 92093-0412

[5] The Novo Nordisk Center for Biosustainability at the University of California San Diego School of Medicine, University of California, San Diego, La Jolla CA 92093-0412

[6] Department of Pediatrics, University of California, San Diego, La Jolla CA 92093-0412

This PDF file includes

Description of Additional Files

Supplementary Text

Figs. S1-S5

References (1-18)

Other Supplementary Materials for this manuscript includes the following:

Supplementary Data 1 as an MS Excel file, containing data for various analyses performed in the paper

Cancer cell flux state calculation code “code.zip” as a zip file, containing MATLAB implementation.

Table of Contents

Additional Files…………………………………………………………………………… 3

Supplementary Results………………………………………………………………………… 4

Supplementary Methods……………………………………………………………………… 20

Supplementary Discussion…………………………………………………………………… 23

Supplementary References…………………………………………………………………… 25

Additional Files

'Supplementary Data 1.xlsx':

File contains results for a number of computational analyses conducted in the manuscript, as well as collected experimental data from the literature. First worksheet of the file is a table of contents.

'calculate_fluxes.m':

File contains the Matlab implementation of the cancer cell metabolic flux state calculation performed in this work. Requires previous installation of the COBRA Toolbox[1] and a linear programming solver such as Gurobi (http://www.gurobi.com/). The most recent version of Gurobi 5 is recommended, which is free for academic use. Code is commented for help. Other required files are contained in code.zip in the Supplenentary Materials.

Supplementary Results

Analysis of alternate objective functions for estimating the metabolic flux states of the NCI60 cell lines

A typical cellular objective assumed for flux balance analysis is the maximization of cellular growth rate. However, since the growth rates were already measured for the NCI60 cell lines, we needed to determine a secondary objective that would provide the best estimate of the cellular metabolic flux state. We tested several plausible objectives: 1) maximizing cytosolic ATP production by maximizing the artificial reaction ATP[c] + H_2O[c] -> ADP[c] + P_i[c] + H[c], 2) maximizing cytosolic NADH production by maximizing the artificial reaction NADH[c] -> NAD[c] + H[c], 3) maximizing cytosolic NADPH production by maximizing the artificial reaction NADPH[c] -> NADP[c] + H[c], 4) minimizing the Euclidean norm of the flux vector subject to the other constraints already set on the model, and 5) maximizing mitochondrial NADPH production by maximizing the artificial reaction NADPH[m] -> NADP[m] + H[m] (Supplementary Data). We evaluated these objectives based on the agreement of the predicted fluxes and 13C data for the MCF7 and A549 cell lines[27,28]. We found that maximizing mitochondrial NADPH production gave the best agreement, while the other candidate objectives failed to reproduce key features of the measured flux states, including correct malate-aspartate shuttle flux and oxygen consumption rates (Supplementary Data).

Alternative hypotheses for principles underlying the Warburg effect

Supplementary Figure 1 shows results referenced in the main text regarding various hypotheses for principles underlying the Warburg effect. We examined a number of hypotheses, including

the need for production of biomass precursors (Supplementary Figure 1a), and various aspects of protein crowding and protein synthesis limitation (Supplementary Figure 1b,c).

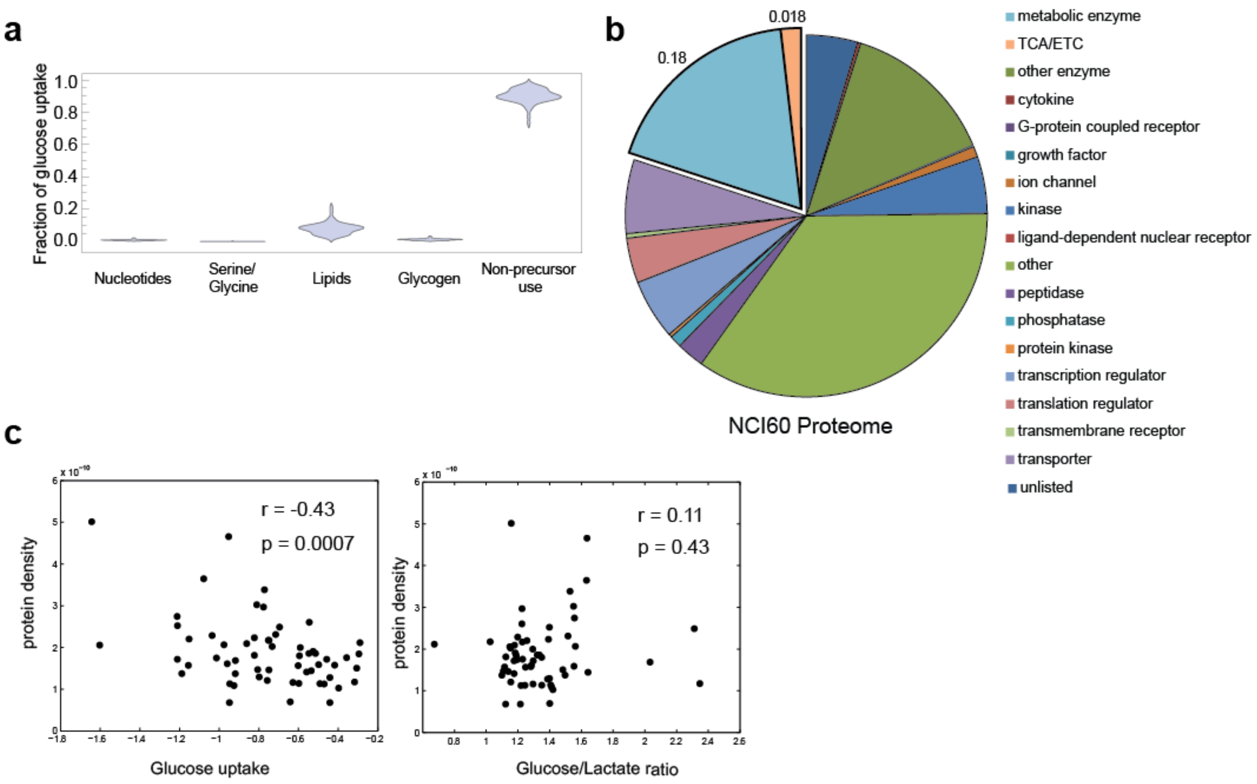

Supplementary Figure 1: Analysis of alternative hypotheses for principles underlying the Warburg effect. a) Fraction of glucose uptake going to biomass precursor and non-precursor production. The majority of glucose goes to energy production, ending up as CO_2. b) Analysis of the average proteome among the NCI60, broken down by functional classification. It is seen that the fraction of metabolic protein involved in oxidative phosphorylation (the TCA cycle and electron transport chain) is small, suggesting that only a marginal amount of protein cost and/or space is saved by shifting from an oxidative to anaerobic energy production phenotype. c) Evaluation of protein crowding metrics vs Warburg effect metrics. No correlation is observed between the protein density and degree of the Warburg effect, quantified by the glucose/lactate ratio. However, dense lines take up greater amounts of glucose, which is the opposite trend that

would be expected if the Warburg effect was a response to minimize the proteome due to macromolecular crowding constraints.

Construction and evaluation of genome-scale cell line-specific models

Using the GIMME algorithm[2] and gene[3] and protein[4] expression data for the NCI60 cell line panel, we initially constructed cell line-specific models for the NCI60 cell line panel from the global human reconstruction Recon 2[5]. Briefly, the GIMME algorithm attempts to find a flux state that satisfied measured growth and metabolite uptake/secretion constraints, while using as few reactions called “Absent” in the gene expression data as possible. If a reaction is active in the flux solution but is not expressed, we say that this reaction is “required in the model” but this flux is inconsistent with the expression data. More details are described in the manuscript on the GIMME algorithm[2].

Growth[6] and metabolite uptake data[7] as well as cell sizes[8] were used to constrain this algorithm. We found that presence in the proteomics data was a good predictor of presence in gene expression data, with a specificity of 0.96, indicating concordance between the data types used in model construction (Figure S2e). However, proteomics data only showed a specificity of 0.06 for presence calls in gene expression data. This low specificity is likely a result of both genes that are transcribed but are not translated as well as limitations in experimental proteomics methods.

Metabolic similarities and differences can be determined from these models, as can the value of integrating multiple data types to achieve a more complete and consistent picture of the

metabolic state of the cell lines. Transcriptomic data showed that expression certain pathways, notably central metabolism and vitamin metabolism, were highly conserved across the NCI60 cell lines, with greater than 90% of reactions having measured gene expression in the majority of cell lines (Figure S2b). This result suggests that the core metabolic network is largely shared among the NCI60 lines, a finding that is somewhat at odds with prior NCI60 model construction efforts that did not include physiological data[9], which we define as growth rate, cell size, and metabolite uptake and secretion data. It should be noted that physiological data has been shown to be more accurate in quantitatively determining metabolic flux maps than expression profiling data that should be considered semi-quantitative[10]. Amino acid metabolism, however, contained notably more reactions with low expression in a subset of cell lines, indicating metabolic variance within these pathways. Notable reactions in Recon 2 with no measured gene expression in certain cell lines included malic enzyme (11 lines), methylthioadenosine phosphatase (11 lines), argininosuccinate synthase (8 lines), the previously mentioned phosphoglycerate dehydrogenase (6 lines), and pyruvate decarboxylase (4 lines).

After running the GIMME algorithm, reaction content becomes more diversified between the cell lines (Figure S2c). This indicates that although the majority of reactions may be consistently expressed between cell lines, the expression penalty for certain absent reactions may cause entire pathways to be shut off in particular cell lines, as long as these pathways are not forced to be active by growth or measured uptake/secretion requirements. This appears to be a concerning feature of the behavior of the GIMME algorithm. For 88 reactions (Figure S2d), metabolite exchange profiles and growth rate data (call functional or physiological data) indicated reactions were active in certain cell lines even though gene expression data was low, highlighting the benefit of incorporating independent data sets in the model construction process.

Notable cases of active but low expressed reactions included Complex II and III from the ETC, several reactions around the urea cycle including ornithine transaminase and argininosuccinate synthase, and the serine synthesis reaction phosphoglycerate dehydrogenase.

After running the GIMME algorithm, we attempted to evaluate and curate the resulting cell-specific models. Specifically, we looked at cases of inconsistencies between the constraints due to growth and metabolite uptake and secretion, and the gene expression data. We term reactions that are expressed ("Present") but cannot carry flux due to constraints "Down-regulated", while reactions that are not expressed ("Absent") but are forced to carry flux by constraints "Up-regulated" (Supplementary Data). We attempted to manually curate these cases to determine whether a 'fix' could be made, such as a modification to the constraints, or addition of a possible previously unknown reaction, or identification of a problematic gene expression probe, to resolve the inconsistency. However, this effort ended up being largely unsuccessful. For illustrative purposes, we include these lists in the Supplementary Data.

From this model curation effort, we decided that the due to the fact that many of the most interesting reaction "Absences" in the expression data were required to be "Present" by growth or metabolite exchange data, little clear value was obtained through the use of these models, as reaction absence called may not be fully trustable. For this reason, we decided to construct a high confidence core metabolic model that was both highly expressed and overlapped with available 13C data that could be used for validation of flux solutions. We also note that our efforts to use cell line-specific models to reproduce the same 13C data were largely unsuccessful.

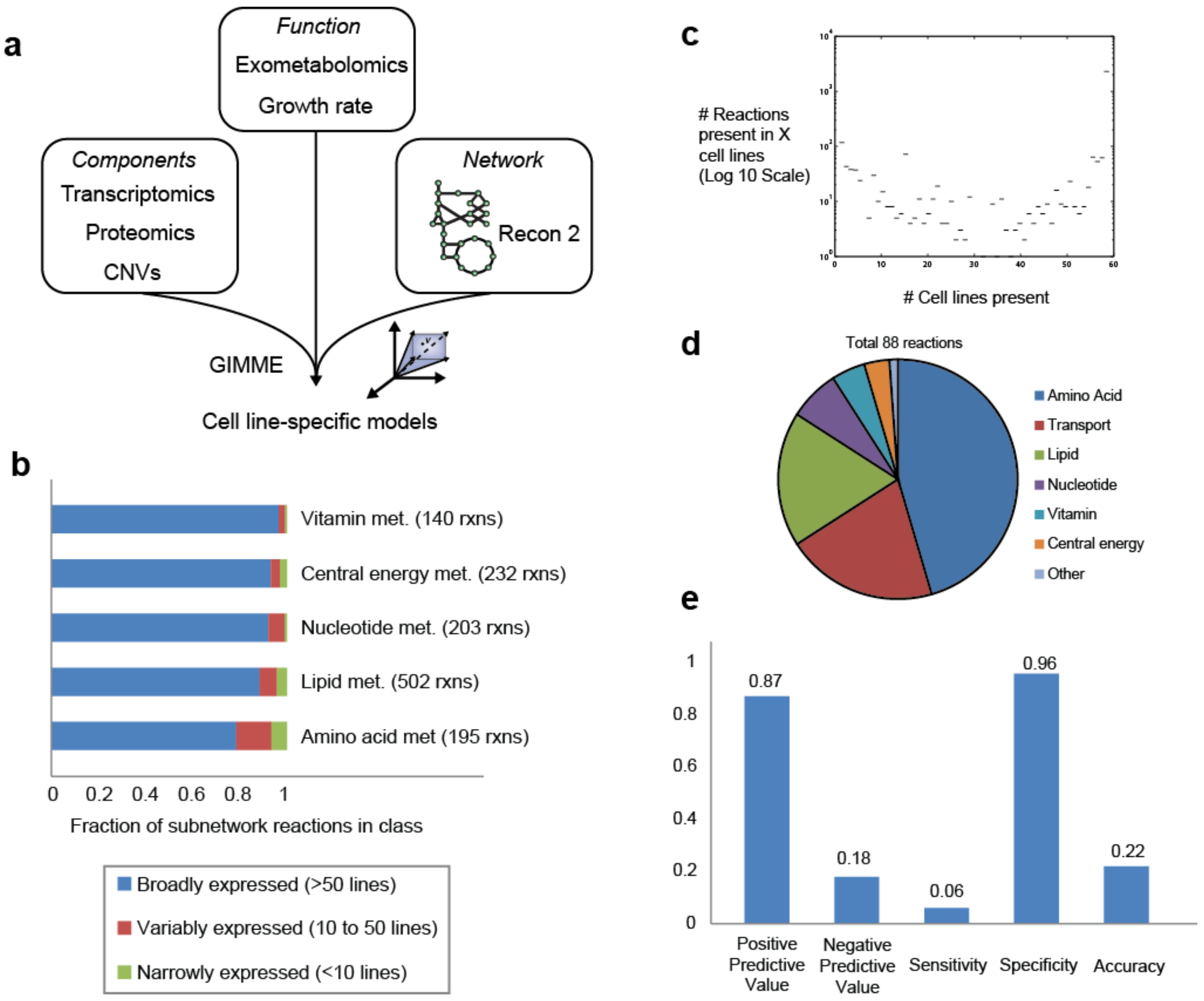

Supplementary Figure 2: Construction and evaluation of cell line-specific genome-scale metabolic models of the NCI60 cell lines. a) Workflow for the construction of cell line-specific genome-scale metabolic models. Gene expression, protein expression, and copy number variation data were integrated with the latest human reconstruction Recon 2 using the GIMME algorithm[2]. b) Analysis of reaction content among cell line-specific models. Vitamin and central energy metabolism were highly expressed across all lines, indicative of the fact that these pathways are necessary for growth. However, amino acid metabolism was more variably

expressed, possibly due to several alternative biosynthetic and catabolic routes being connected to many amino acids. c) Log plot of reaction presence across the NCI60 cell lines following cell-specific model construction with the GIMME algorithm, indicating variable reaction content between cell lines. d) Analysis of inconsistent reactions among the NCI60 cell lines, where a reaction was not expressed in the gene expression data but was required to be present in the GIMME constraint-based modeling algorithm in order to satisfy growth or metabolite exchange constraints. e) Statistical comparison of the agreement of gene and protein expression data. The calculation of the terms is described in the Table of Contents of the Supplementary Data in the "Gene-Protein Stats" table. The high positive predictive value indicates that when protein is present, the gene is very likely to be called as expressed in the gene expression data as well, indicating good agreement in the positive calls between data sets.

Analysis of protein content

Protein contents for the NCI60 have been measured[8], but issues with the data create complications with the direct use of the data. First, correlation with measured cell volumes is relatively low (Pearson $R^2 = 0.23$). However, cell volumes were measured in replicate through a microscopy based approach, while protein contents were measured with a single sample per cell line. Second, when integrated with metabolite uptake data[7] and growth rate data[6], both of which were measured in replicate, 23 lines were predicted to not be able to sustain the measured protein content, with several lines substantially deficient. We define the sustainable protein content as the mass of protein that a cell could produce given measured metabolite uptakes and growth rate. It is a value calculated using flux balance analysis and identifies the largest mass/cell value that allows a feasible flux balance analysis solution constrained by measured metabolite uptakes and growth rate for a particular cell line

First we verified that the protein levels were physically consistent with measured cell volumes assuming a protein density of 1.35 g/ml[11]. There was an average protein volume fraction of 0.15, and no volume fraction of protein was higher than 0.4, suggesting that no physical volume constraints were likely to be violated by either the measured or sustainable protein contents. We then examined potential causes of this discrepancy between sustainable and measured protein content. Three possible explanations we identified were that the cells were catabolizing the excess of amino acids for energy, the cells were using the excess amino acids for protein synthesis of secreted protein that would not have been measured, or the cells were using the excess amino acids for protein synthesis of internal proteins and thus the measured protein content were erroneous. Notably, the observation that many lines did not take up enough amino acids to sustain their measured protein is already suggestive of erroneous protein data. To

examine the alternatives, we looked at correlations between expression of genes involved in catabolism of essential amino acids as well as genes encoding secreted proteins with the amount of excess amino acids taken up. We observed no widespread correlation of expression of amino acid catabolic genes with excess amino acids taken up, which did not support the idea that an excess of amino acids are taken up.

Instead, we proceeded with the hypothesis that some of the measured protein data was erroneous and sought to correct the data using information from the amount of protein that the cells could sustain production of during growth, and using the measured cellular volumes as validating data. This hypothesizes that the minimum amount of amino acid possible is catabolized or removed from the cell as secreted protein. This hypothesis was evaluated by comparing the amount of protein sustainable above the measured protein content, termed the observed excess sustainable protein (Supplementary Data, "Protein Corrected" table), with the gene and protein expression for both 1) enzymes lying within essential amino acid catabolic pathways, or 2) coding for secreted protein. The list of amino acid catabolic genes was derived from Recon 2 (Supplementary Data). The list of secreted proteins was derived from UNIPROT by identifying proteins tagged with the "secreted" term (Supplementary Data). No correlation was observed between expression of these genes and the observed excess sustainable protein, supporting the notion that this excess sustainable protein is actually being synthesized into internal protein and should be added to the cellular protein content.

To calculate the sustainable protein for use as the cell protein, we performed an FBA optimization to maximize the cell mass subject to measured metabolic uptake and secretion, growth rate, and biomass composition. Cell line-specific biomasses were not taken into account, but previous studies measuring biomass composition found very similar compositions across cell

lines and mammalian organisms[12-14], suggesting this is not a large source of error. The procedure used is described in the Methods of the main text. The final agreement between corrected protein and measured cellular volumes was noticeably better than for uncorrected protein (Pearson R^2 = 0.60 vs R^2 = 0.23) (Supplementary Fig. 3e).

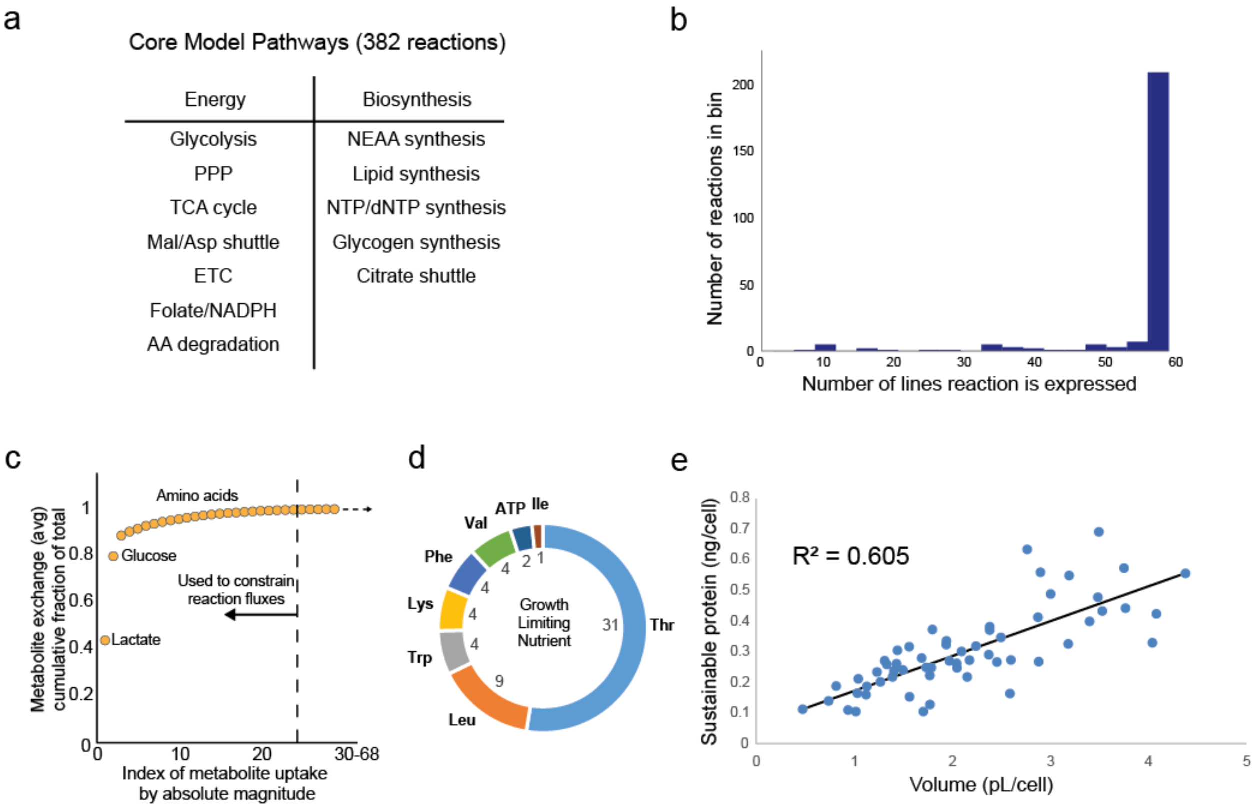

Supplementary Figure 3: Summary of core cancer metabolic model and analysis of protein content. a) Summary of the metabolic pathways and functions represented by the core cancer metabolic network, consisting of 382 reactions. b) Expression calls for reactions in the core model. As is seen, the vast majority of reactions in the core are called as expressed, supportive of the definition of the model as a core cancer network. c) Cumulative distribution plot of absolute metabolite uptakes from a published data set[7]. Twenty-three of the highest flux metabolites were used as constraints on the core model (Supplementary Data 1), representing over 99% of the absolute metabolite exchange flux by mass. d) The limiting growth nutrient among the NCI60 cell lines. The majority of lines are limited by the availability of a particular essential amino acid,

while only two lines are limited by energy production. e) Correlation between the corrected intracellular protein estimation and measured cell volume.

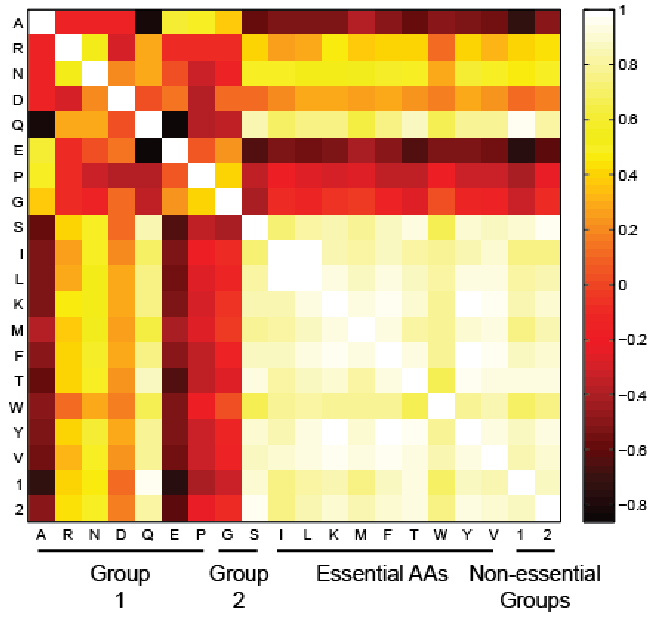

Supplementary Figure 4: Correlation among amino acid uptakes in the NCI60 cell line panel. Amino acids are grouped by common biosynthetic pathway. Group 1 consists of amino acids most closely tied to glutamine uptake. Group 2 consists of amino acids most closely tied to serine uptake. It is seen that by summing amino acids by groups, the net uptake of each group (the Non-essential Groups columns) are highly correlated with essential amino acid uptakes, even more so than serine and glutamine individually.

NADPH production and consumption in the NCI60 cell lines

One additional analysis we performed was to examine the role of the redox cofactors produced by the pathways associated with the high uptake metabolites glucose and serine. We found that growth-associated NADPH demands are dominated by fatty acid and steroid metabolism (Supplementary Fig. 5a), and are met by significant contributions to the cytosolic NADPH pool by the pentose phosphate pathway (66%) and folate metabolism (34%), which is in the range of previous estimates[15] (Supplementary Fig. 5b). Notably, a large majority of this NADPH production (mean 86%) originates from glucose. Thus, cytosolic NADPH production pathways meet the growth requirement of NADPH for the cell and are powered by glucose uptake.

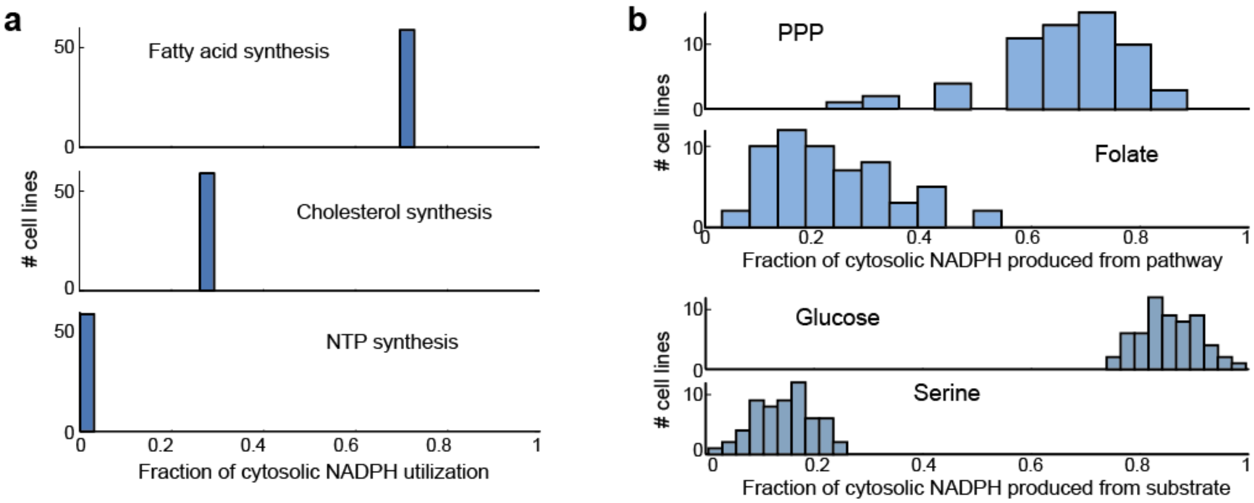

Supplementary Figure 5: Cytosolic NADPH balance. a) Cytosolic NADPH utilization, showing dominance of fatty acid and steroid synthesis. b) Cytosolic production of NADPH. The pentose phosphate pathway and folate pathway are both significant contributors, with the pentose phosphate pathway predicted to produce an average of 66% of cytosolic NADPH. However, an even higher fraction of cytosolic NADPH (86%) is glucose-derived rather than serine derived, due to additional NADPH from the glucose-driven *de novo* serine synthesis pathway.

Analysis of correlation between transporter isozyme expression differences and metabolite uptake flux differences

One auxiliary analysis we performed that was of interest was to examine whether cell line-specific transport isozymes had a noticeable correlation with measured metabolite exchanges. We performed a targeted analysis of transporter kinetics for glucose and lactate, selected due to their high transport flux and degree of annotation of transporters. For each of these metabolites, a large number of transporter isoforms exist (over 15 for glucose, 4 for lactate). We were interested in determining whether the choice of transporter isoforms is associated with a particular metabolic phenotype. We found that, for glucose, expression of the most kinetically efficient glucose transporter, GLUT3, is significantly correlated with glucose uptake (0.0303 Wilcoxon rank sum), suggesting alternate transporter expression as a potential factor underlying differences in growth scaling. However, no association was found for lactate transporter expression.

Supplementary Methods

Normalization and curation of expression data

The construction of cell-specific models was performed using gene expression data. Triplicate gene expression microarray data for the NCI60 was normalized using GCRMA. Gene presence absence calls used in cell line-specific model construction were generated as follows. We found that applying a general cutoff for presence worked poorly, based on the ability of cells to satisfy a growth state given the available metabolite uptakes. We attribute this failure to probe-specific bias, which we also observed, when multiple probes were present for the same gene. Instead, we curated each probe and used the presence of triplicate data and fact that expression data was generated in a single study for the NCI60 lines in order to manually assess probe-specific bias to create presence/absence calls. GCRMA yielded a floor of 2.23 and a ceiling above 10. Based on this value, and the distribution of values across all sample, we created the following classification system.

1) Any probe with no cell line having a triplicate mean greater than 3 was called as "no signal"

2) Certain probes where the triplicate variance was high relative to the mean was called as no signal

3) Where signal was observed (low triplicate variance), and the values were relatively continuous between cell lines with no apparent break or signal floor, all cell lines were called "present"

4) Where signal was observed (low triplicate variance), but there was a clear discontinuity with some lines having 'high' expression and others 'low' expression, a

cutoff was assigned between the two groups, and the probe for the lower groups was called "absent"

5) When multiple probes were present for the same gene, probes were sorted by dynamic range (max-min) and averaged if correlated with the probe with greatest dynamic range, otherwise uncorrelated lower dynamic range probes were deleted

The normalized and curated data with expression value cutoffs is available in the Supplementary Data.

Correction of ^{13}C tracing data for comparison to calculated fluxes

^{13}C tracing data for the MCF7[16] and A549[17] cell lines was corrected prior to comparison to calculated fluxes. These values were only corrected for the purpose of comparison to calculated fluxes, but these corrections were not subsequently used to modify the calculated fluxes in any way for later analyses. Details of the calculations can be found in the Supplementary Data.

The A549 cell line data was corrected to account for a substantial difference in measured lactate secretion between the ^{13}C study and the exometabolomic study[7]. The difference between the two values was used to correct the LDH reaction as well as the reactions affected by the difference in leftover pyruvate from glycolysis, which consists of PDH and PC as well as downstream TCA reactions. As PDH and PC are alternate routes to enter the TCA cycle, they were corrected in the same 90:10 split that was used as a constraint in the flux balance calculations performed in this work.

The MCF7 cell line data was corrected to account for a difference in malate-aspartate shuttle usage between the approximate model used in the ^{13}C study, which did not have NADH as a metabolite and combined malate, oxaloacetate, and aspartate into a single metabolite MOA (Table 2 in the cited paper), and our fully described reconstruction. The version of cytosolic malic enzyme in their manuscript converts the cytosolic aggregate metabolic MOA to pyruvate. However, their aggregate malate-aspartate shuttle only accounts for the net loss of this malate pool toward pyruvate, it does not account for the need to shuttle NADH produced by glycolysis into the mitochondria for oxidation. The amount of NADH produced by glycolysis agrees between the ^{13}C data and our flux calculations and lactate production is insufficient to regenerate NAD at steady-state. Our model is constrained by this NADH production in a way that the approximate model used in the ^{13}C study is not. The consequence is that our model calculates that the active form of malic enzyme is the mitochondrial form, which is the canonical active form[18], rather than the cytosolic form as reported in the ^{13}C study. It is unknown whether placing a correct NADH balance constraint on the model used in the ^{13}C study would cause their flux solution to prefer the mitochondrial malic enzyme form. Still, operating on the assumption that the NADH balance issue is sufficient reason to doubt the accuracy of the fluxes involving metabolites affected by the malate-asparate shuttle, we corrected the ^{13}C data as follows. We calculated the difference between the mitochondrial and cytosolic malic enzyme fluxes and used this value to adjust the fluxes affected by the active malic enzyme being mitochondrial. These reactions were isocitrate dehydrogenase, the malate-aspartate shuttle, and PDH and PC. PDH and PC were corrected by this value in the same 90:10 split that was used as a constraint in the flux balance calculation performed in this work.

Supplementary Discussion

The role of metabolic models in providing a structure for data analysis

The quantitative analysis of the required use of the metabolic network to provide the cellular resources (biosynthesis and energy) needed to produce the observed physiological demands reveals a relatively simple basis for observed characteristics of cancer cell metabolism. The regularization of all the data types is based on systems level balancing of mass and energy requirements for observed cellular functions provides a useful framework for the analysis and understanding of the hallmarks of observed metabolic functions in well characterized cancer cell lines. This study shows that disparate data sets on the NCI60 collection of cancer cell lines can be integrated and reconciled using genome-scale models as a context. This approach discovered the overall structure of metabolism in cancer cells appears to be very simple and stoichiometrically determined. It is characterized by two groups of amino acids, the serine and essential amino acid groups, being taken up at net levels primarily set by the protein synthesis demand. Meanwhile, each dominant uptake metabolite is taken up in excess in a manner that fuels production of NADPH and/or ATP above biosynthetic requirements, coupled to an overflow of metabolites at the end of corresponding production pathways but prior to a mitochondrial catabolic pathway.

Gene expression data curation and consistency with measured growth and metabolite exchanges

There appear to be three possible explanations for the observation that many reactions would be called ‘off’ by expression considerations alone, but are forced to be on to allow a flux state consistent with measured metabolite exchange profiles and growth rates. The first option is

that the lowly expressed reaction is not active, and that instead an alternate pathway exists that is providing the metabolic function. Second, it is possible that the reaction is active but the protein catalyzing the reaction is different from the annotated protein, suggesting unknown isozymes exist. Given the large number of cases observed and their location in highly studied pathways, it appears unlikely that such a large number of unknown alternate pathways and/or isozymes exist. The third possibility is that the reaction is active and the annotated protein is present but in low amounts that are sufficient for catalysis. We believe this last case to be the true case, which raises a potential concern with model construction methods based solely on expression data but lack metabolite uptake/secretion profiles and growth rates.

Supplementary References

1 Schellenberger, J. *et al.* Quantitative prediction of cellular metabolism with constraint-based models: the COBRA Toolbox v2.0. *Nat Protoc* **6**, 1290-1307, doi:10.1038/nprot.2011.308 (2011).

2 Becker, S. A. & Palsson, B. O. Context-specific metabolic networks are consistent with experiments. *PLos Comp Biol* **4**, e1000082 (2008).

3 Shankavaram, U. T. *et al.* CellMiner: a relational database and query tool for the NCI-60 cancer cell lines. *BMC Genomics* **10**, 277, doi:10.1186/1471-2164-10-277 (2009).

4 Moghaddas Gholami, A. *et al.* Global proteome analysis of the NCI-60 cell line panel. *Cell reports* **4**, 609-620, doi:10.1016/j.celrep.2013.07.018 (2013).

5 Thiele, I. *et al.* A community-driven global reconstruction of human metabolism. *Nat Biotechnol* **31**, 419-425, doi:10.1038/nbt.2488 (2013).

6 O'Connor, P. M. *et al.* Characterization of the p53 tumor suppressor pathway in cell lines of the National Cancer Institute anticancer drug screen and correlations with the growth-inhibitory potency of 123 anticancer agents. *Cancer Res* **57**, 4285-4300 (1997).

7 Jain, M. *et al.* Metabolite profiling identifies a key role for glycine in rapid cancer cell proliferation. *Science* **336**, 1040-1044, doi:10.1126/science.1218595 (2012).

8 Dolfi, S. C. *et al.* The metabolic demands of cancer cells are coupled to their size and protein synthesis rates. *Cancer Metab* **1**, 20, doi:10.1186/2049-3002-1-20 (2013).

9 Yizhak, K. *et al.* A computational study of the Warburg effect identifies metabolic targets inhibiting cancer migration. *Mol Syst Biol* **10**, 744, doi:10.15252/msb.20134993 (2014).

10 Machado, D. & Herrgard, M. Systematic evaluation of methods for integration of transcriptomic data into constraint-based models of metabolism. *PLoS Comput Biol* **10**, e1003580, doi:10.1371/journal.pcbi.1003580 (2014).

11 Erickson, H. P. Size and shape of protein molecules at the nanometer level determined by sedimentation, gel filtration, and electron microscopy. *Biol Proced Online* **11**, 32-51, doi:10.1007/s12575-009-9008-x (2009).

12 Sheikh, K., Forster, J. & Nielsen, L. K. Modeling hybridoma cell metabolism using a generic genome-scale metabolic model of Mus musculus. *Biotechnol Prog* **21**, 112-121, doi:10.1021/bp0498138 (2005).

13 Altamirano, C. *et al.* Analysis of CHO cells metabolic redistribution in a glutamate-based defined medium in continuous culture. *Biotechnol Prog* **17**, 1032-1041, doi:bp0100981 (2001).

14 Bonarius, H. P. *et al.* Metabolic flux analysis of hybridoma cells in different culture media using mass balances. *Biotechnol Bioeng* **50**, 299-318, doi:10.1002/(SICI)1097-0290(19960505)50:3<299::AID-BIT9>3.0.CO;2-B (1996).

15 Fan, J. *et al.* Quantitative flux analysis reveals folate-dependent NADPH production. *Nature* **510**, 298-302, doi:10.1038/nature13236 (2014).

16 Forbes, N. S., Meadows, A. L., Clark, D. S. & Blanch, H. W. Estradiol stimulates the biosynthetic pathways of breast cancer cells: detection by metabolic flux analysis. *Metab Eng* **8**, 639-652, doi:S1096-7176(06)00062-0 (2006).

17 Metallo, C. M., Walther, J. L. & Stephanopoulos, G. Evaluation of 13C isotopic tracers for metabolic flux analysis in mammalian cells. *J Biotechnol* **144**, 167-174, doi:10.1016/j.jbiotec.2009.07.010 (2009).

18 DeBerardinis, R. J. *et al.* Beyond aerobic glycolysis: transformed cells can engage in glutamine metabolism that exceeds the requirement for protein and nucleotide synthesis. *Proc Natl Acad Sci U S A* **104**, 19345-19350, doi:0709747104 (2007).
